# Dietary Habits and Nutritional Knowledge of Adolescents in Lower Silesia (Poland): A Comparative Study Between 2011 and 2023

**DOI:** 10.3390/nu18071066

**Published:** 2026-03-26

**Authors:** Paulina Kokoszka, Tomasz Lesiów, Malgorzata Agnieszka Jarossová

**Affiliations:** 1 Department of Agri-Engineering and Quality Analysis, Wroclaw University of Economics and Business, 53-345 Wroclaw, Poland; 2 Faculty of Commerce, Bratislava University of Economics and Business, 852-35 Bratislava, Slovakia

**Keywords:** nutrition, primary school students, survey research, dietary habits, nutritional knowledge

## Abstract

Background: Adolescence is a critical developmental period during which dietary habits are formed and may influence long-term health outcomes. Monitoring changes in adolescents’ eating behaviors and nutrition-related knowledge over time is important for developing effective health promotion strategies. The aim of this study was to compare adolescents’ (Lower Silesia, Poland) dietary habits and nutritional knowledge between two study periods (2011 and 2023) using comparable survey methods. Methods: A repeated cross-sectional comparison of two independent cohorts was conducted using an identical questionnaire in both study periods. The 2023 cohort included 14-year-old primary school students (*n* = 100; 48 girls and 52 boys), while the comparison group consisted of adolescents aged 13–15 years assessed in 2011 (*n* = 377; 202 girls and 175 boys). Anthropometric measurements and self-reported data on dietary habits and nutritional knowledge were analyzed using descriptive statistics and group comparison tests. Results: The findings indicate changes in selected dietary behaviors and levels of nutritional knowledge among adolescents over the studied period. A higher percentage of students in 2023 reported eating four meals per day and obtaining information about healthy eating from the Internet rather than from television. Students in 2023 were also more likely to recognize the relationship between diet and attention, identify the harmful effects of energy drinks and excessive fast-food consumption, and provide correct answers regarding proper nutrition. Nutritional knowledge improved over time, with a mean percentage of correct responses of 71.9% in 2023 compared with 63.7% in 2011. Although nutritional awareness improved in several areas, certain unhealthy eating habits remained prevalent, including irregular breakfast consumption and frequent intake of sweets. Changes in the distribution of body weight categories were also observed, with gender-specific differences between cohorts. Conclusions: The results suggest that improvements in nutritional knowledge alone may not be sufficient to ensure positive changes in dietary behavior among adolescents. Continued monitoring of adolescent nutrition and the development of comprehensive health promotion strategies addressing both knowledge and environmental influences remain necessary.

## 1. Introduction

Proper nutrition is crucial for the health and well-being of children and adolescents [1,2]. Over the last two decades, an increasing number of adolescents and young adults have developed unhealthy eating habits, and their level of knowledge is often insufficient to support the adoption of appropriate dietary practices [3,4].

Numerous publications in both Polish and international literature report that the percentage of individuals with obesity continues to increase [4,5,6,7,8,9]. The consequences of obesity may include diseases such as diabetes, dental caries, hypertension, musculoskeletal disorders, and cancer, as well as disability in adulthood and premature death [6,10,11]. Almost 26% of Polish children attending primary school have difficulty maintaining a healthy body weight [6], and this percentage continues to rise. According to a study conducted by the Food and Nutrition Institute, this trend is influenced by reduced physical activity and poor eating habits, often acquired at home and at school [6].

Poor eating behaviors among young people are primarily associated with insufficient nutritional awareness. They are also influenced by parental nutritional knowledge [12,13,14]. In some cases, however, the relationship is reversed, and a high level of parental nutritional knowledge may coexist with overweight and obesity in children [15]. The situation is more complex among preschool children, as parents’ nutritional knowledge is supported by nurseries and kindergartens [16]. Moreover, many young people are influenced by fashion trends and advertising in their everyday shopping choices and often do not verify product information, for example, by reading food labels [17]. The energy and nutritional requirements of children and adolescents vary according to age, sex, weight, height, body composition, and nutritional and emotional status. However, excessive consumption of sweets or fast food does not have beneficial effects in any of these groups [18]. This issue is compounded by the fact that highly processed foods are often palatable, quick to prepare, and have become a marker of a fast-paced lifestyle, thereby further increasing the risk of weight gain and obesity [19].

Adolescence is a critical period for the development of dietary habits that may persist into adulthood and influence long-term health outcomes. Unhealthy eating behaviors established during this stage of life are associated with an increased risk of overweight, obesity, and diet-related chronic diseases later in life. For this reason, understanding adolescents’ dietary patterns and their level of nutrition-related knowledge is an important component of public health strategies aimed at improving population health.

Monitoring changes in adolescents’ dietary behaviors over time remains challenging because many studies rely on comparisons with previously published research using different methodologies, questionnaires, or study populations. Such differences often limit the ability to identify consistent trends in adolescent nutrition. Studies that use comparable research tools across different time points may therefore provide more reliable insights into potential generational changes in dietary habits and nutrition-related knowledge.

Therefore, the aim of the present study was to compare adolescents’ dietary habits and nutritional knowledge between two study periods (2011 and 2023) using the same questionnaire. By analyzing two independent cohorts of adolescents surveyed more than a decade apart, this study seeks to identify potential differences in eating behaviors, nutrition-related knowledge, and selected anthropometric indicators among adolescents.

## 2. Materials and Methods

### 2.1. Methodology

A repeated cross-sectional comparison of two independent cohorts was conducted using an identical questionnaire in both study periods. The 2023 cohort included eighth-grade students aged 14 years attending the Maria Konopnicka Primary School in Jaworzyna Śląska (Lower Silesia Province, Poland). The research tool was a paper-based questionnaire. A total of 100 completed questionnaires were collected (48 girls and 52 boys). The study was approved by the school teachers’ council, the parents’ council, and the headmaster. Moreover, it received approval from the Rector’s Commission on Research Ethics of Wrocław University of Economics and Business (No. 36/2025, 1 September 2025).

**Figure 1:**
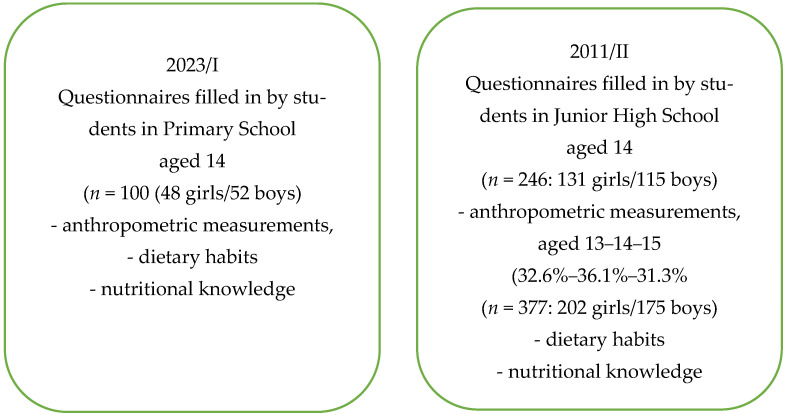
The experimental design. Junior high school attendance followed seven years of primary school education. In the 2022–2023 school year, an educational reform extended primary school education to eight years.

In the 2023 study, anonymous questionnaires were delivered to the headmaster, who distributed them to students, who completed them during the homeroom period (Figure 1). In the introductory section, each respondent provided their height, weight, gender, and age. The survey consisted of 18 questions assessing dietary habits and food preferences related to the consumption of selected food groups, followed by 10 questions evaluating nutritional knowledge. The questionnaire included closed-ended questions, with predefined answer options.

In the second stage, the results obtained in 2023 were compared with data collected by Kosiorowska in 2011 among students from three junior high schools (No. 10, 11, and 16) in Wrocław [5]. Respondents were aged 13–15 years (*n* = 377; 202 girls and 175 boys). Anthropometric data were obtained from school health recorders, and dietary habits were assessed using a structured questionnaire. The questionnaires were approved by the school teachers’ council, the parents’ council, and the headmaster.

In both studies (2011 and 2023), the questionnaires were pilot-tested on a small group of students to ensure clarity, comprehensibility, and appropriateness of response options.

Although the age ranges were not identical, both cohorts represent mid-adolescence, a developmentally comparable stage characterized by increasing autonomy in dietary choices.

The experimental design is presented in Figure 1.

Body mass index (BMI) was calculated as weight (kg) divided by height squared (m^2^). BMI values were compared with age-specific percentile charts for boys and girls developed by Palczewska and Niedźwiedzka in 2001 [20]. The prevalence of underweight, healthy weight, and excessive body weight was assessed.

The cut-off points for deficiency, healthy weight, and excessive body weight differed slightly from those presented by the World Health Organization Reference Curves [21]. However, the criteria used in the present study, according to Palczewska and Niedźwiedzka [20], enabled direct comparison with data from 2011.

Snacks were defined as additional food consumed between main meals.

### 2.2. Statistical Analysis

The responses obtained were analyzed in terms of the following parameters:

- Mean, minimum, maximum, and median values of height, body weight, and BMI by gender;
- Prevalence of underweight, healthy weight, and excessive body weight among students in 2023;
- Average BMI of primary school students;
- Responses regarding knowledge of proper nutrition.

The sample size was *n* = 100. Assuming a 20% proportion of the studied characteristic, an estimation error of 8%, and a 95% confidence level (*u_α_* = 1.96), the minimum required sample size was 96 individuals. The value was calculated from the following equation [22,23]:

(1) $$n\;=\;{\left(u_{\alpha }\right)}^{2}\;(p(1-p))/e^{2}$$

where *n*—minimum sample size, *p*—population proportion (0.20), *e*—estimation error (8%), and *u_α_*—standard normal deviate (1.96).

*n* = 1.96^2^ (0.2 × 0.8)/0.08^2^, *n* = 0.614656/0.0064, *n* = 96

Differences between groups were assessed using Student’s *t*-test for normally distributed variables or the Mann–Whitney U test for non-normally distributed variables, as well as the chi-square (χ^2^) test. Fisher’s exact test was applied when expected cell counts were small. A two-sided *p*-value < 0.05 was considered statistically significant.

Given the independent cohort design and differences in age structure between 2011 and 2023, multivariable regression models were not applied. The analysis focused on descriptive and comparative assessment of generational trends rather than causal inference.

All analyses were performed using MS Excel 2010 (Microsoft Corporation, Redmond, WA, USA) [22].

## 3. Results

### 3.1. Height, Weight, and Body Mass Index (BMI) of Adolescents

Table 1 presents descriptive statistics for height, body weight, and body mass index (BMI) stratified by gender.

Boys had significantly greater body height (*p* = 0.0135) and body weight (*p* = 0.0415) than girls (Table 1). No statistically significant difference in BMI was observed between boys and girls (*p* = 0.0776).

Table 2 presents the distribution of underweight, healthy body weight, and excessive body weight among primary school students.

Boys had a lower percentage of healthy body weight (51.9%) compared with girls (68.7%) (Table 2). Excessive body weight was more common among boys than among girls (19.2% vs. 6.3%). Moreover, 9.6% boys were classified as having lower-than-healthy body weight. The difference between sexes approached statistical significance (χ^2^ test, *p* = 0.0514). Due to the limited number of participants with excessive body weight (n = 13), multivariable logistic regression analysis was not performed.

Overall, 60% of students had a healthy body weight, including 19% classified as having upper healthy body weight and 5% lower healthy body weight. Excessive body weight was observed in 13% of the total sample.

### 3.2. Dietary Habits of Primary School Students in 2023

**Figure 2:**
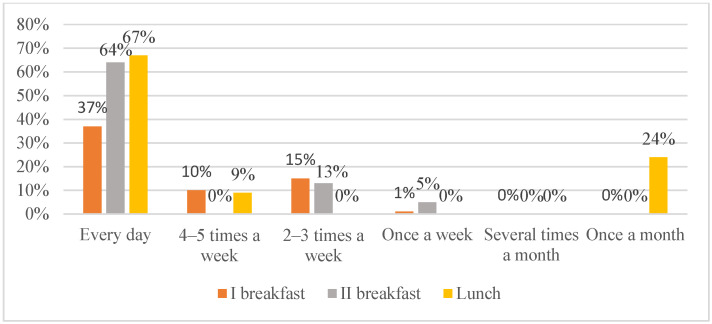
Frequency of consumption of first breakfast, second breakfast, and lunch among primary school students in Jaworzyna Śląska in 2023.

Only 37% of students reported eating breakfast at home every morning (Figure 2), while the same proportion declared not eating breakfast at home at all. Ten percent consumed breakfast 4–5 times per week, 15% 2–3 times per week, and 1% once per week. Among the reasons for skipping breakfast, 81% indicated a lack of appetite, whereas 19% preferred to sleep longer. No significant sex differences were observed in daily breakfast consumption (girls 37.5% vs. boys 36.5%, *p* > 0.05).

Most students (64%) brought a second breakfast to school every day; however, 18% never did so. Thirteen percent reported bringing it 2-3 times per week, and 5% only once per week. Boys were significantly more likely than girls not to bring breakfast to school or to do so only once per week (18% vs. 5%, *p* = 0.017).

Regarding lunch consumption at home, 67% of respondents reported eating lunch at home daily, 24% occasionally, and 9% 4–5 times per week (Figure 2).

It was found that 11% of students consumed only 1–2 meals per day, 70% reported eating 3–4 meals, and 17% consumed 5–6 meals daily. Regarding fluid intake, 58% reported drinking 1.5–2 L of water daily, 9% consumed 3 L or more, 25% drank 1 L, and 8% drank less than 0.5 L per day. Snacking between main meals was common. The most frequently consumed snacks were sweets (43%), followed by fruit and vegetables (21%) and salty snacks (19%). The least frequently selected snacks were dairy drinks (4%) and carbonated beverages (1%). Only 11% reported not snacking between meals.

Regarding purchases from the vending machine or the school shop, 27% reported never making such purchases. No student declared buying items daily; 5% did so several times per week, 14% several times per month, 15% once per month, and 39% occasionally. The most common reason for purchasing food at school was insufficient food brought from home (53%). Additionally, 31% responded not feeling hungry in the morning and therefore not bringing breakfast. Seven percent preferred shop or vending machine food because they considered it tastier, and 9% indicated peer influence.

Fast food consumption was widespread: 39% consumed fast food several times per month, 38% once per month, and 1% daily or several times per week. Twenty percent consumed it occasionally, and 1% reported not consuming fast food at all. The most popular fast food items were chips (54%), followed by kebabs (20%) and burgers (12%). The least popular were hot dogs (7%), wraps (5%), and casseroles (2%). The main reason for consuming fast food was convenience (quick preparation) (64%), followed by better taste compared with healthy food (14%). Twelve percent reported a lack of time, and 10% indicated peer influence. Boys were significantly more likely than girls to consume fast food frequently (daily, several times per week, or several times per month (29% versus 12%, *p* = 0.0164).

Regarding physical activity, 34% of students exercised daily, 48% several times per week, 16% several times per month, 1% occasionally, and 1% not at all. Significant gender differences were observed. Boys engaged in sports approximately twice as often as girls. However, paradoxically, a higher percentage of boys reported participating in sports several times per month or less frequently compared with girls (14% vs. 4%, *p* = 0.0286). Regular physical activity (≥several times per week) was slightly more common among boys (67.3%) than girls (60.4%), although the difference was not statistically significant (*p* > 0.05).

The declared frequency of consumption of selected food groups is presented in Table 3.

Most students consumed fruit (49%), light bread (45%), and mineral water (85%) daily (Table 3). Fewer students consumed meat (35%) and milk and dairy drinks (32%) daily. Several times per week, students consumed cheese (53%), milk and dairy drinks (45%), meat (44%), cold cuts (39%), pasta (42%), raw vegetables (40%), boiled vegetables (36%), and eggs (36%). Groats and rice were chosen less frequently (20%), while fish and seafood were eaten only a few times per month by 25% of respondents.

The relatively high intake of sweets (46%), salty snacks (50%), and carbonated drinks (32%) indicates an unhealthy dietary pattern, as these products were consumed several times per week.

No significant differences were observed in the frequency of consumption of most food groups, except carbohydrate beverages. Boys were less likely than girls to declare consuming carbohydrate beverages once per month or less frequently (8% vs. 18%, *p* = 0.0302).

Despite satisfactory levels of nutritional knowledge (Section 3.3), the frequent consumption of sweets, salty snacks, and sugar-sweetened beverages suggests a discrepancy between knowledge and dietary behavior.

### 3.3. Nutritional Knowledge of Primary School Students in 2023

Most students obtained nutritional information from the Internet (62%), followed by parents (24%) and physicians (7%). Only 3% cited television and 4% teachers as their primary source of information. Regarding self-assessment, 45% of students rated their knowledge of healthy eating as good, 36% as sufficient, 11% as moderate, and 8% as very good. In addition, 55% of respondents perceived their body weight as appropriate, while 28% considered themselves slightly overweight, and 13% underweight; 2% indicated high underweight and 2% severe overweight.

Most students (84%) correctly recognized the harmful health effects of energy drinks, whereas 16% did not share this view. Almost all respondents (94%) agreed that excessive fast food consumption negatively affects health, while only 6% disagreed. Furthermore, 84% acknowledged that inadequate nutrition adversely affects attention and concentration, while 16% disagreed.

Seventy-three percent of respondents agreed that daily breakfast consumption is necessary, while 27% did not consider it essential. Regarding recommended meal frequency, 69% indicated 3–4 meals per day and 31% selected 5–6 meals per day. In terms of daily fluid intake, 70% of students correctly identified 1.5–2 L as appropriate, 24% selected 2.5–3 L, and 6% selected 3.5 L or more.

Almost all respondents (99%) recognized that practicing sports positively affects health, with only 1% holding the opposite view. Regarding knowledge of diet-related diseases, 69% of students correctly identified conditions such as dental caries, hypertension, mental disorders, osteoporosis, and cancer as being associated with improper nutrition, whereas 31% provided incorrect responses.

When asked about the meaning of the Healthy Eating Pyramid, 42% of respondents correctly indicated that it presents dietary recommendations developed by specialists. In contrast, 36% associated it with the proportions of macronutrients in the diet, and 22% interpreted it as guidance for composing a daily menu. Significantly fewer boys than girls identified it as representing principles of food composition (6% vs. 16%, *p* = 0.0203).

Overall, the average percentage of correct responses on the nutritional knowledge assessment was 71.9%.

### 3.4. Anthropometric Measurements of Primary School Students in 2011 and 2023

Anthropometric characteristics of students assessed in 2011 (Table 4) and 2023 (Table 1) were comparable and did not differ significantly (*p* < 0.05). However, boys in 2023 were, on average, 1.7 kg heavier and 2 cm taller than those in 2011.

A higher percentage of girls had a healthy body weight in 2023 (68.7%, Table 2) than in 2011 (45%, Table 5). Among boys, the percentages were similar (51.9% and 51.4%). A lower body weight deficiency was less common in 2023 than in 2011 among both girls (4.2% versus 5.3%) and boys (1.9% versus 3.5%). A favorable trend was observed among girls, where the percentage of excessive body weight decreased by 13.6% (from 19.9% to 6.3%). In contrast, among boys, the prevalence of excessive body weight increased by 5.4% (from 13.8% to 19.2%).

Overall, the proportion of students with healthy body weight increased from 48% in 2011 to 60% in 2023. However, these findings should be interpreted with caution due to sex-specific differences and potential contextual factors between the two time points.

### 3.5. Dietary Habits of Primary School Students in 2011 and 2023

**Figure 3:**
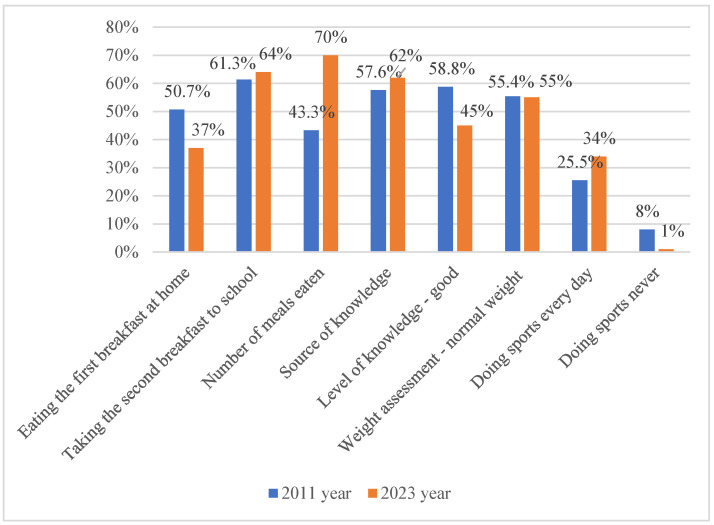
Comparison of eating habits (responses 1–3) and nutritional knowledge (responses 4–8) among primary school students in 2011 and 2023.

Based on comparable questions from a 2011 survey conducted by Kosiorowska [5], the proportion of students eating breakfast at home before school decreased from 50.7% in 2011 to 37% in 2023 (Figure 3).

The percentage of students bringing a second breakfast to school increased from 61.3% in 2011 to 64% in 2023. These findings may suggest that parents and carers should remain actively involved in ensuring adequate daily nutrition.

A substantial increase was observed in the proportion of students consuming four meals per day, rising from 43.3% in 2011 to 70% in 2023. Despite this improvement in meal frequency, snacking between meals remained common in both study periods. In 2011, students most frequently chose fruit (75.1%) and sweets (71.3%), followed by yogurts (51.2%) and salty snacks (23.6%) (multiple-choice question). In 2023, the most commonly reported snacks were sweets (43%), fruit and vegetables (21%), and salty snacks (19%).

These findings indicate that although some aspects of dietary patterns have improved, the high frequency of consumption of energy-dense, nutrient-poor foods remains a persistent feature of adolescents’ eating behaviors.

### 3.6. Nutrition Knowledge of Primary School Students in 2011 and 2023

In 2011, television (57.6%) was the main source of nutritional information, whereas in 2023, the Internet (62%) became the dominant source (Figure 3).

In 2011, 58.8% of students rated their nutritional knowledge as good; in 2023, this proportion decreased to 45%. Despite this decrease in self-perception, approximately 55% of students in both years reported having a healthy body weight. Positive changes were observed in physical activity patterns. Daily participation in sports increased from 25.5% to 34% in 2023, while the proportion of students not participating in sports decreased from 8% to 1% (Figure 3).

**Figure 4:**
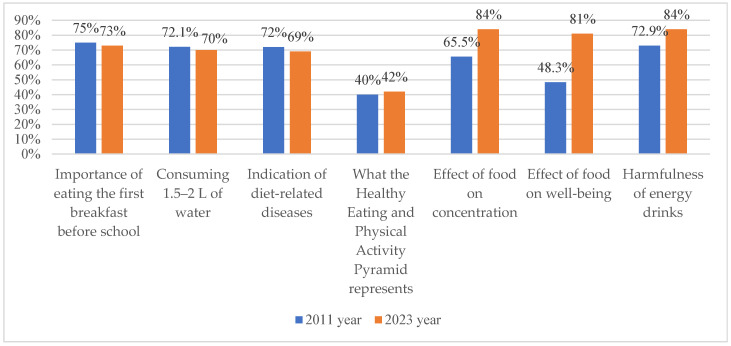
Comparison of young people’s knowledge about proper nutrition in 2011 and 2023.

In terms of specific knowledge areas, 75% of students in 2011 and 73% in 2023 recognized the importance of daily breakfast consumption (Figure 4). Similarly, 72.1% of students in 2011 and 70% in 2023 correctly identified 1.5–2 L as the recommended daily fluid intake. The proportion of students correctly identifying diet-related diseases decreased slightly from 72% in 2011 to 69% in 2023. Knowledge regarding the Healthy Eating Pyramid increased from 40% to 42%. Substantial improvements were observed in awareness of the relationship between diet and cognitive as well as psychological functioning. The proportion of students recognizing the impact of diet on concentration increased from 65.5% in 2011 to 84% in 2023, while recognition of its effect on well-being increased from 48.3% to 81%. Similarly, awareness of the harmful effects of energy drink consumption increased from 72.9% in 2011 to 84% in 2023.

Overall, the mean proportion of correct responses increased from 63.7% in 2011 to 71.9% in 2023 (Figure 4), indicating an improvement in objective nutritional knowledge over time. However, these improvements were not consistently reflected in dietary behaviors, suggesting a persistent gap between knowledge and practice.

## 4. Discussion

The present study compared two independent cohorts of adolescents surveyed at different time points using the same questionnaire, allowing a cross-cohort assessment of potential generational differences in dietary habits and nutritional knowledge. Although such a design does not allow causal inference or tracking of individual behavioral changes over time, it provides valuable insights into population-level tendencies in adolescent nutrition.

The dietary habits of primary school students have evolved over the analyzed period, likely reflecting broader societal transformations such as digitalization, accelerated lifestyles, changes in food availability, and post-pandemic behavioral shifts.

A particularly concerning finding is the decline in daily breakfast consumption, which decreased from 50.7% in 2011 to 37% in 2023. Breakfast plays a critical role in supporting cognitive performance, metabolic regulation, and overall development during adolescence. A high prevalence of breakfast skipping may negatively affect concentration and academic performance. Previous studies have emphasized the importance of eating breakfast before school, highlighting its role in providing essential energy and nutrients required for proper physical and cognitive functioning during adolescence [24,25]. Breakfast consumption has also been shown to positively influence cognitive outcomes, including attention, memory, learning abilities, and academic achievement in school-aged children [25]. Furthermore, systematic reviews indicate that skipping breakfast is associated with a poorer diet quality and a higher prevalence of unhealthy weight status among youth, reinforcing the importance of regular meal patterns in obesity prevention [26]. Our findings are consistent with those reported by Cisińska [27], who observed that 39.5% of junior high school students in the Łódzkie Voivodeship did not regularly consume breakfast before school.

These findings highlight the important role of parents in promoting regular breakfast consumption. Teachers, particularly during early morning classes, may also contribute by emphasizing the health and cognitive consequences of skipping breakfast. Structural factors such as early school start times, long commuting distances, and changing family routines may further contribute to breakfast omission. In addition, adolescents increasingly exercise autonomy in food-related decisions, and breakfast skipping may reflect time constraints or weight-control behaviors rather than simple negligence.

Although the proportion of students consuming four meals per day increased to 70% in 2023, meal frequency alone does not guarantee dietary quality. The persistently high intake of sweets (43%) and salty snacks (19%) suggests that a higher number of meals may coexist with suboptimal food choices. Several factors may have contributed to the increase in the proportion of students consuming four or five meals per day, including parental influence, school-based educational programs, peer group norms, easier access to food outlets, the wide availability of food products, and adolescents’ growing ability to interpret nutritional information on food labels. Consuming four or five meals per day supports balanced energy distribution and healthy development. Importantly, meals provided both at home and within school food services should remain varied and nutritionally balanced, incorporating fruits, vegetables, protein sources, complex carbohydrates, and healthy fats [28].

Despite ongoing educational efforts, increasing daily fruit and vegetable consumption while reducing the intake of sweets and salty snacks remains challenging. Encouraging adolescents to replace energy-dense, nutrient-poor products with healthier alternatives such as fruits (e.g., apples, oranges, bananas) and vegetables (e.g., baby carrots) continues to be difficult. Although 49% of students reported daily fruit consumption in 2023—compared with 33.4% reported by Cisińska [27]—snacking patterns remain unfavorable. Fruit and vegetable snacks (21%) and salty snacks (19%) were still chosen less frequently than sweets (43%). While students and their parents are generally aware that fruits and vegetables provide vitamins, minerals, dietary fiber, and bioactive compounds [29], sweets high in sugar continue to be widely consumed due to taste preferences and easy availability. These findings suggest that taste preferences, accessibility, and environmental exposure may outweigh nutritional knowledge in shaping food choices.

In this context, promoting healthy dietary behaviors through broader media campaigns, improved availability of healthier snack options, and increased awareness of the benefits of fruit consumption as a snack alternative may be beneficial [30].

A notable shift was observed in the sources of nutritional information. The Internet has replaced television as the primary source, with 62% of students relying on digital media in 2023. Although digital platforms facilitate access to information, they also expose adolescents to contradictory or misleading content, highlighting the importance of digital literacy education. Schools play a central role in this process, as they are responsible not only for delivering knowledge but also for teaching students how to critically evaluate information and distinguish reliable content from misinformation [31]. Although students demonstrated a satisfactory mean knowledge score (71.9%), self-assessed knowledge declined compared with 2011 (from 58.8% to 45%). This discrepancy may reflect either greater critical awareness or confusion resulting from exposure to diverse and sometimes conflicting online content. Therefore, digital literacy and critical appraisal skills should be considered essential components of school-based nutrition education.

The literature consistently emphasizes the pivotal role of parents in shaping children’s dietary behaviors [32]. However, in the present study, only 24% of students identified parents as their primary source of nutritional knowledge, which is comparable to the 20% reported by Cisińska [27]. Teachers, physicians, and nurses accounted for less than 10% of reported sources. This pattern may reflect increasing independence during adolescence, but it may also indicate limited parental engagement in structured nutrition communication. Additionally, the marginal role of teachers and health professionals (<10%) suggests underutilization of institutional channels for evidence-based health promotion. These findings indicate a need to strengthen the involvement of health professionals and educators in nutrition education. In Poland, ongoing educational reforms, including initiatives aimed at increasing access to school psychologists and nutrition specialists, may help improve this situation.

Encouragingly, adolescents demonstrated greater awareness of the relationship between diet, concentration, and overall well-being. Recognition of the harmful effects of energy drinks also increased. Greater awareness may support more informed food choices; however, perceptions do not automatically translate into sustained behavior change, particularly in environments where unhealthy products remain easily accessible and heavily marketed.

Gender-specific differences were also observed. Although the overall proportion of students with a healthy body weight increased, excessive body weight decreased among girls but increased among boys (Table 2). These divergent trends may reflect differences in lifestyle behaviors, social influences, or attitudes toward body image during adolescence, suggesting the need for gender-sensitive prevention strategies.

Maintaining a healthy body weight remains essential for long-term health [1,33], yet only around half of students perceived their body weight as being within the normal range. Misperception of body weight may influence both unhealthy dietary restraint and a lack of corrective action. Consequently, interventions should aim to promote accurate self-assessment while avoiding stigmatization and the risk of disordered eating behaviors.

An increase in regular physical activity was also observed. Numerous studies have examined the relationship between BMI and physical activity among children and adolescents, highlighting the importance of integrating regular exercise into daily life as part of a healthy lifestyle [34,35,36,37]. Physical activity is a fundamental component of health promotion and should complement a balanced diet [38].

Interpretation of temporal differences should consider broader societal changes occurring between 2011 and 2023, including increased exposure to digital media and lifestyle shifts following the COVID-19 pandemic. Because the study used an independent cohort design, the findings represent generational tendencies rather than causal longitudinal changes.

An improvement in nutritional knowledge was also observed, with the mean percentage of correct responses increasing from 63.7% to 71.9%. Awareness of the harmful effects of energy drinks and fast food also increased. However, these improvements were only partially reflected in dietary behaviors. For example, irregular breakfast consumption and frequent intake of sweets remained relatively common. This observation highlights the widely reported gap between nutrition knowledge and actual dietary practices among adolescents [39,40].

Adolescents’ food choices are influenced not only by knowledge but also by environmental availability, peer norms, taste preferences, marketing exposure, convenience, and emotional factors. According to Flores et al. [41], educational interventions grounded in behavioral theories may improve dietary outcomes more effectively than knowledge-only approaches, underscoring the need for multi-component strategies. Future interventions should therefore move beyond traditional didactic education toward comprehensive, environment-oriented, and policy-supported strategies that address both cognitive and structural determinants of adolescent nutrition.

Monitoring dietary habits and nutritional knowledge among primary school students remains essential. Educational efforts by parents, teachers, and nutritionists should extend beyond knowledge transmission and aim to foster positive attitudes toward healthy eating. Adolescents are more likely to adopt and maintain healthy behaviors when they understand their relevance and feel personally engaged. Without such involvement and awareness of the consequences of dietary choices, long-term behavioral change is unlikely.

Failure to prevent obesity and diet-related health problems should not be attributed solely to adolescents. Responsibility is shared among families, educational institutions, health professionals, and broader societal systems operating at the national level. Sustainable improvements in adolescent nutrition, therefore, require coordinated, multisectoral action.

### Strengths and Limitations of the Study

The principal strength of this study lies in the direct comparison of two independent adolescent cohorts (2011 vs. 2023). An additional methodological advantage is the use of an identical questionnaire across both survey periods, which enhances the comparability and internal consistency of the collected data. The findings highlight the importance of integrating nutrition education with broader environmental and behavioral interventions when addressing adolescents’ dietary habits.

Nevertheless, several limitations should be acknowledged. First, the age composition of the cohorts differed: the 2011 cohort included adolescents aged 13–15 years, whereas the 2023 cohort consisted exclusively of 14-year-old students. Age-related differences may influence both nutritional knowledge and eating behaviors, as older adolescents typically demonstrate greater autonomy in food choices and may experience different levels of exposure to social and environmental influences. These factors may confound direct comparisons between cohorts; therefore, the results should be interpreted as reflecting generational patterns rather than precise longitudinal changes.

Second, substantial social, technological, and cultural changes occurred between 2011 and 2023, including increased exposure to social media, evolving school food policies, and the long-term lifestyle consequences of the COVID-19 pandemic. Because these external factors were not directly assessed in the present study, their potential influence on dietary behaviors and health knowledge cannot be separated from genuine temporal trends in cross-cohort comparisons.

Third, the study design does not allow longitudinal tracking at the individual level. Although comparisons of independent cross-sectional cohorts can identify population-level tendencies, they do not permit causal inference or the assessment of within-individual behavioral changes over time. As a result, conclusions regarding behavioral evolution should be interpreted with appropriate caution, as cross-sectional designs inherently limit the ability to establish temporal or causal relationships between exposures and outcomes.

Additionally, several external determinants known to influence dietary behavior and nutritional knowledge were not assessed in this study. These include socioeconomic status, food security, parental education level, and characteristics of the home and school food environments. The absence of these variables limits the ability to control for potential confounding factors and may reduce the generalizability of the findings.

Previous research indicates that nutrition education alone, without supportive environments and policy-level interventions, has a limited impact on long-term behavioral change. For example, Kosiorowska [5] demonstrated that although school-based nutrition education combined with the removal of unhealthy food options initially improved eating behaviors, these effects diminished over time. These findings indicate that knowledge gains may not be maintained in the absence of continued environmental support. Consequently, improvements in nutritional knowledge should be interpreted cautiously when behavioral outcomes remain suboptimal.

In summary, this study provides insight into changes in adolescent nutrition knowledge and self-reported dietary behaviors over time; however, the results should be interpreted with caution. The limitations outlined above reflect common methodological challenges in nutrition epidemiology, including cohort design constraints, environmental influences, and the difficulty of isolating determinants of behavioral change. Future research incorporating multivariable modeling and longitudinal follow-up designs would help clarify the observed trends and better account for potential confounding influences.

Despite these limitations, continued monitoring of adolescents’ dietary behaviors using comparable research instruments remains essential for identifying emerging patterns and supporting the development of effective public health nutrition strategies.

## 5. Conclusions

Significant temporal changes in adolescents’ dietary habits and nutritional knowledge were observed between 2011 and 2023.

Positive trends included improved nutritional knowledge, increased awareness of the harmful effects of energy drinks, and greater recognition of the relationship between diet, concentration and overall well-being. However, the frequent consumption of sweets, fast food, and other energy-dense foods remains common, suggesting that improvements in knowledge alone are insufficient to produce substantial changes in dietary behavior.

The increase in the mean proportion of correct responses (from 63.7% to 71.9%) did not consistently translate into healthier dietary practices, highlighting a persistent gap between knowledge and behavior.

Continuous monitoring of adolescents’ dietary behaviors remains essential for the development of effective public health nutrition strategies.

Achieving sustainable behavioral change among adolescents requires comprehensive, multi-level interventions that address both cognitive and structural determinants of dietary behavior. The present findings suggest that long-term improvements depend on continuous, systematic, and integrated strategies extending beyond traditional classroom-based education. Effective interventions should combine school-based nutrition education with environmental modifications, active parental and caregiver engagement, policy measures regulating food availability, and broader community support.

Future prevention strategies should therefore integrate educational, environmental, and family-based components. Given the independent cohort design, the present findings reflect generational tendencies rather than causal longitudinal effects. Future studies should employ longitudinal cohort designs and account for potential confounding variables such as socioeconomic status, parental practices, access to food environments, and media exposure.

In summary, although improvements in nutritional knowledge and risk awareness are encouraging, they are not sufficient on their own. Meaningful and sustained behavioral change among adolescents requires comprehensive, context-sensitive approaches that address both the cognitive and structural determinants of dietary choices.

## Figures and Tables

**Table 1:** Minimum, maximum, and mean values and medians of height, weight, and body mass index (BMI) among primary school students in 2023.

Sex	N	Body Height (cm)				Body Weight (kg)				BMI (kg/m^2^)			
		Mean, Sd	Median	Min.	Max.	Mean, Sd	Median	Min.	Max.	Mean	Median	Min.	Max.
Girls	48	161.8 ^a^ ± 5.43	161.5	150	171	53.3 ^a^ ± 10.78	50.0	38	97	−20.2 ^a^ ± 3.17	19.5	14.6	33.1
Boys	52	165.6 ^b^ ± 9.09	165.0	161	178	56.4 ^a^ ± 12.82	55.0	47	88	20.4 ^a^ ± 3.60	19.6	15.6	31.1
Total	100	163.7 ± 7.79	163.25	150	178	54.85 ± 11.99	52.5	38	97	20.3 ± 3.40	19.55	14.6	31.1

Source: Own study. Means marked with the different letters within columns differ significantly (*p* < 0.05).

**Table 2:** The incidence of deficiency, healthy and excessive body weight among primary school students in 2023.

Students of Primary School in Jaworzyna Śląska						
BMI Centile	Girls		Boys		Altogether	
	*n* = 48	(%)	*n* = 52	(%)	*n* = 100	(%)
<3–emaciation	1	2.1	0	0	1	1
from 3 to 10–body weight deficiency	1	2.1	1	1.9	2	2
**Total values indicating body weight deficiency**	**2**	**4.2**	**1**	**1.9**	**3**	**3**
from 10 to 25–lower healthy body weight (thinness)	0	0	5	9.6	5	5
**from 25 to 75–healthy body weight**	**33**	**68.7**	**27**	**51.9**	**60**	**60**
from 75 to 90-upper healthy body weight	10	20.8	9	17.3	19	19
from 90 to 97–overweight	1	2.1	7	13.4	8	8
>97–obesity	2	4.2	3	5.8	5	5
**Total values indicating excessive body weight**	**3**	**6.3**	**10**	**19.2**	**13**	**13**

Source: Own study.

**Table 3:** Frequency of consumption of selected food groups by students of the primary school in Jaworzyna Śląska in 2023.

	Every Day	Several Times a Week	Several Times a Month	Once a Month	Less Often	Never
(%)						
Milk and milk beverages	32	45	14	0	6	3
Cheeses	21	53	12	3	6	5
Raw vegetables	13	40	21	7	12	7
Boiled vegetables	14	36	29	5	12	4
Fruit	49	42	4	1	4	0
Meat	35	44	18	1	0	2
Cold cuts	26	39	17	3	4	11
Fishes and seafood	2	6	25	16	25	26
Eggs	3	36	37	8	10	6
Light bread	45	29	13	1	6	6
Dark bread	17	26	28	4	16	9
Margarine	8	14	23	15	20	20
Groats and rice	5	20	45	17	8	5
Pasta	9	42	45	0	1	3
Sweets	30	46	17	0	6	1
Salty snacks	13	50	27	6	4	0
Carbonated drinks	18	32	24	8	14	4
Mineral water	85	10	2	1	1	1
Tea and coffee	44	37	15	1	3	0

Source: Own study.

**Table 4:** Minimum, maximum, and mean values, as well as medians, of height, weight, and body mass index (BMI) among primary school students in 2011 [5].

Sex	N	Body High (cm)				Body Weight (kg)				BMI (kg/m^2^)			
		Mean, Sd	Median	Min.	Max	Mean, Sd	Median	Min.	Max.	Mean, Sd	Median	Min.	Max.
Girls	131	162.2 ^a^ ± 6.71	163.0	137	180	54.5 ^a^ ± 11.35	52.7	35.8	97	20.6 ^a^ ± 3.81	19.9	15.0	34.8
Boys	115	163.6 ^a^ ± 9.17	164.0	137	186	54.7 ^a^ ± 12.32	53.0	32.0	90	20.3 ^a^ ± 3.32	19.4	14.6	31.2
Total	246	162.9 ± 7.96	163.0	137	186	54.61 ± 11.79	53.0	32.0	97	20.47 ± 3.59	19.81	14.6	34.8

Source: Own study. Means marked with the same letters within columns do not differ significantly (*p* < 0.05).

**Table 5:** The incidence of deficiency, healthy and excessive bodyweight among primary school students in 2011 [5].

Students of Primary School in 2011						
BMI Centile	Girls		Boys		Altogether	
	n = 131	(%)	n = 115	(%)	n = 246	%
<3–emaciation	0	0	1	0.9	1	0.4
from 3 to 10–body weight deficiency	7	5.3	3	2.6	10	4.1
**Total values indicating body weight deficiency**	**7**	**5.3**	**4**	**3.5**	**11**	**4.5**
from 10 to 25–lower healthy body weight (thinness)	16	12.2	12	10.4	28	11.4
**from 25 to 75–healthy body weight**	**59**	**45.0**	**59**	**51.4**	**118**	**48**
from 75 to 90-upper healthy body weight	23	17.6	24	20.9	47	19.1
from 90 to 97–overweight	9	6.9	8	6.9	17	6.9
>97–obesity	17	13	8	6.9	25	10.1
**Total values indicating excessive body weight**	**26**	**19.9**	**16**	**13.8**	**42**	**17**

Source: Own study.

## Data Availability

Data are contained within the article.
